# Supplementary material for: Race (black-white) and sex inequalities in tooth loss: A population-based study
Source: PLoS One. 2022 Oct 13;17(10):e0276103. doi: 10.1371/journal.pone.0276103 (PMC9560604; doi:10.1371/journal.pone.0276103)
Supplement: S1 Table — (DOCX) [file pone.0276103.s001.docx]

Supplementary Table 1. Sociodemographic characteristics according to sex and race, in the population aged 10 or over, ISACamp 2014-2015.

| Characteristic |  | | Woman (%) | | | |  | p* |  | Man (%) | | | |  | p* |
| --- | --- | --- | --- | --- | --- | --- | --- | --- | --- | --- | --- | --- | --- | --- | --- |
|  | Black | | |  | White | |  |  | Black | |  | White | |  |  |
|  | N | % | | IC95% | n | % | IC95% |  | n | % | IC95% | n | % | IC95% |  |
| Age |  |  | |  |  |  |  | **0.005** |  |  |  |  |  |  | **0.002** |
| 10 to 19 years old | 211 | 41.3 | | (34.8-48.1) | 287 | 58.7 | (51.9-65.1) |  | 239 | 45.9 | (40.5-51.4) | 272 | 54.0 | (48.5-59.4) |  |
| 20 to 39 years old | 112 | 35,2 | | (28.4-42.6) | 175 | 64.8 | (57.4-71.6) |  | 101 | 37.2 | (30.3-44.7) | 149 | 62.7 | (55.2-69.6) |  |
| 40 to 59 years old | 88 | 30.7 | | (24.2-38.1) | 166 | 69.3 | (61.9-75.8) |  | 57 | 26.3 | (18.9-35.4) | 148 | 73.6 | (64.6-81.1) |  |
| 60 years old or over | 147 | 25.8 | | (21.2-30.9) | 432 | 74.2 | (69.1-78.8) |  | 107 | 28.2 | (20.9-36.8) | 271 | 71.8 | (63.2-79.1) |  |
| Total | 558 |  | |  | 1060 |  |  |  | 504 |  |  | 840 |  |  |  |
| Income in MW |  |  | |  |  |  |  | **0.000** |  |  |  |  |  |  | **0.000** |
| <1 | 320 | 44.1 | | (37,7-50.6) | 390 | 55.9 | (49.4-62.3) |  | 277 | 47.9 | (38.8-57.0) | 283 | 52.1 | (42.9-61.1) |  |
| ≥1 and ≤3 | 213 | 29.1 | | (24.4-34.4) | 538 | 70.9 | (65.6-75.7) |  | 201 | 29.3 | (23.6-35.6) | 442 | 70.7 | (64.3-76.3) |  |
| > 3 | 25 | 12.3 | | (6.7-21.6) | 131 | 87.7 | (78.4-93.3) |  | 21 | 14.5 | (7.8-25.3) | 109 | 85.5 | (74.6-92.1) |  |
| Schooling (years) |  |  | |  |  |  |  | **0.001** |  |  |  |  |  |  | **0.001** |
| 0-4 | 154 | 37.7 | | (29.9-46.1) | 321 | 62.3 | (53.8-70.1) |  | 117 | 39.7 | (29.3-51.1) | 198 | 60.3 | (48.8-70.6) |  |
| 5-8 | 193 | 42.9 | | (36.1-49.9) | 253 | 57.1 | (50.1-63.9) |  | 185 | 40.8 | (33.3-48.7) | 251 | 59.2 | (51.2-66.6) |  |
| 9 or more | 211 | 27.5 | | (21.9-33.8) | 486 | 72.5 | (66.2-78.0) |  | 202 | 29.6 | (24.0-35.7) | 390 | 70.4 | (64.2-75.9) |  |
| District |  |  | |  |  |  |  | **0.006** |  |  |  |  |  |  | **0.002** |
| East | 47 | 13.7 | | (7.0-25.0-26-5) | 203 | 86.2 | (74.9-92.9) |  | 28 | 12.4 | (3.9-33.0) | 145 | 87.6 | (66.9-96.1) |  |
| North | 87 | 31.9 | | (20.8-45.6) | 181 | 68.0 | (54.4-71.3) |  | 133 | 47.7 | (35.5-60.0) | 142 | 52.3 | (39.9-64.4) |  |
| Northwest | 117 | 41.3 | | (28.7-55.1) | 187 | 58.7 | (44.9-80.3) |  | 59 | 22.8 | (16.0-31.3) | 161 | 77.1 | (68.6-83.9) |  |
| Southwest | 127 | 35.5 | | 27.4-44.74 | 210 | 64.6 | (55.6-72.6) |  | 100 | 35.3 | (26.3-45.3) | 174 | 64.7 | (54.6-73.6) |  |
| South | 180 | 41.2 | | [29.6-53.7) | 279 | 58.8 | (46.3-70.4) |  | 184 | 45.3 | (31.6-59.6) | 218 | 54.7 | (40.4-68.3) |  |
| IPVS (Vulnerability) |  |  | |  |  |  |  | **0.000** |  |  |  |  |  |  | **0.000** |
| The Most Very Low | 20 | 11.5 | | (3.9-28.6) | 88 | 88.5 | (71.6-9762) |  | 5 | 4.9 | (0.9-22.1) | 58 | 95.0 | (77.9-99.0) |  |
| Very Low | 179 | 23.6 | | (17.8-30.7) | 576 | 76.4 | (69.0-82.8) |  | 174 | 24.4 | (17.8-32.5) | 442 | 75.6 | (67.5-82.2) |  |
| Low | 127 | 42.1 | | (35.3-49.2) | 191 | 57.9 | (50.9-64.2) |  | 133 | 43.6 | (35.7-51.7) | 177 | 56.4 | (48.2-64.2) |  |
| Average | 139 | 51.5 | | (38.7-64.1) | 133 | 48.5 | (35.4-61.7) |  | 108 | 50.3 | (35.5-65.0) | 95 | 49.7 | (34.9-64.5) |  |
| High | 33 | 51.4 | | (31.8-70.6) | 32 | 48.6 | (29.2-68.5) |  | 30 | 46.2 | (23.2-70.9) | 28 | 53.8 | (29.0-76.6) |  |
| Very High | 60 | 57.9 | | (41.1-73.0 | 40 | 24.1 | (26.9-58.8) |  | 54 | 62.9 | (38.6-82.0 | 40 | 37.1 | (17.9-61.4) |  |
