# Supplementary material for: Race (black-white) and sex inequalities in tooth loss: A population-based study
Source: PLoS One. 2022 Oct 13;17(10):e0276103. doi: 10.1371/journal.pone.0276103 (PMC9560604; doi:10.1371/journal.pone.0276103)
Supplement: S1 Fig — (DOCX) [file pone.0276103.s002.docx]

Supplementary Figure 1. Percentage of frequency of tooth loss, according to race, by sex, ISACamp 2014/15.
