# Supplementary material for: Race (black-white) and sex inequalities in tooth loss: A population-based study
Source: PLoS One. 2022 Oct 13;17(10):e0276103. doi: 10.1371/journal.pone.0276103 (PMC9560604; doi:10.1371/journal.pone.0276103)
Supplement: Attachment — Submitted filename: Response to Reviewers.docx [file pone.0276103.s003.docx]

**PONE-D-21-32428**

**Race (black-white) and sex inequalities in tooth loss: a population-based study**

**PLOS ONE**

Dear editors

We are submitting the new version of our manuscript. We would like to thank the reviewers for the suggestions, which have certainly enriched our paper. All comments and suggestions were fully taken into account in the preparation of this new version. Our point-by-point responses to the issues raise are listed below:

Review Comments to the Author

Reviewer #1:

1. The authors do not explain the concept of intersectionality in the introduction and do not justify the use of race in biomedical publications. Authors are based on too old references on the topic.
**Response:** We modified the text and added the concept of intersectionality in the introduction: “In this way, it is worth considering the analytical strategy of intersectionality, developed by Crenshaw in 1989 to study the black feminism. Intersectionality, drawing on a set of social issues and perspectives, explain an experience of multiple subordination that cannot be reduced to the simple sum of underprivileged groups [34]”. (Lines 59-63).

We also justify the use of race in biomedical publications: “Racial minorities, in this case black people, may biologically embody the effects of racism [7,12], with everyday (or even less common) discriminatory exposures [12]. Adversities throughout life, such as poverty, psychosocial stresses, stereotypes, and the context of housing, can affect the physical and mental health, altering cardiocirculatory, metabolic and immunological functions [8]. In oral health is possible that inequities are due to poverty [10], levels of education [4] or discrimination in health care [13]. In Brazil, skin color has been studied as proxy of race and this indicator is commonly referred as color/race [14,15,16]. In health, in contrast only to the biologic approach, race may be considered as a concept socially constructed by historic dynamics and power relations [8,12].” (Lines 27-34)

2. The RERI analysis is sound. However, without a proper justification for the outcome (lost of 1 tooth or no – What is the usefulness? ) and a miscellaneous in ordinal category (No loss, loss of 1 tooth, loss of more than one tooth, or loss of all teeth) in complicated to readers understand.

**Response:** Thank you for the comment and I take the opportunity to clarify some points. We added this justification on the text: “Tooth loss is an important marker of oral health, since each tooth is a dental organ and its subtraction has consequences on occlusal adjustment, temporomandibular joint disorder, reduction of the masticatory board and consequently overload for the digestive system [37]. Besides, the loss of at least one tooth since it represents neglect in the dental field, resulting in an increase in the level of severity of oral diseases, in addition to reflecting the model of oral health care adopted and the way individuals understand the condition [38].” (Line 97-103)

Moreover, in the last paragraph of methodology, we complemented: “… Therefore, we decided to focus the primary analysis on the dichotomous outcome, which also represents the most practical way to assess and present interactions [39, 40]. However, on the other hand, the analysis of the same variable as ordinal can show us a gradient of tooth loss, varying its degrees of loss [41]. This analysis was used only to confirm trends in tooth loss in the black population. (Lines 103-106)

3. The DAD used by the authors is difficult to understand. Authors do not put "Place of residence" or "vulnerability index" linked to race. This is a mistake due to the structural factors related to ethnic inequalities. Recent evidence explaining racial inequities put it on evidence (neighbourhood conditions) in oral health (https://pubmed.ncbi.nlm.nih.gov/29634429/). Moreover, the authors did not analyze health behaviours, important mediators explaining tooth loss.
**Response:** We appreciate the comments and the reference indicated and we reviewed the DAG. We understand that neighborhood conditions are important aspects in the health and specifically in oral health. But our thinking about the DAG is the issues of determination. We understand that race is closely linked to places of residence, but these variables are not directly related in a causal diagram. Explicitly, a race does not cause a place of residence, nor a place of residence cause a race. These variables can be associated, as the reviewer correctly indicated, but the only explanation it would be sharing a common cause.

Because can be a long list of potential unmeasured variables, fitting this role of being a common cause of place and race, we follow a common convention that visually simplifies the DAG by representing all unmeasured variables with the same causal structure (i.e., the same arrows in and out) as a single node (Digitale JC, Martin JN, Glymour MM. Tutorial on directed acyclic graphs. J Clin Epidemiol. 2021 Aug 8:S0895-4356(21)00240-7. doi: 10.1016/j.jclinepi.2021.08.001). In this case, we included the concept of “familiarity” as that node representing all common determinants of both, place of residence and race.

In the same sense, vulnerability (as a context variable measured with the IPVS) in which people are born, would neither determine nor be determined by race. But it would be determined by factors such as familiarity and place of residence.

On the other hand, we agree with reviewer regarding the health behaviours as important mediators of demographic determinants. Therefore, we included a variable on the DAG (health behavior) determined by sex, race and age, which would be causally connected with the outcome (Please see new DAG with this inclusion). However, such our objective was to estimate the total effects (not to analyse mediation) of race and sex, specifying their interaction, no additional adjustment was necessary.

During revision of DAG, we perceived that pattern of participation would be better described as a common effect of sex and age than a common cause. So, as it can be conditionate by decision of participate of population, it can imply a collider bias, opening a path. However, this bias it would be also corrected by adjusting by age. Therefore, again, the set of variables indicated to adjusted remain the same. Inserted in the text lines 149-161.

4. The outcome of tooth loss is very strange for me. For population comparisons, the authors do not use international classifications like functional dentition (more than 20 permanent teeth) and severe tooth loss (less than ten remaining teeth).

**Response:** The question used to collect the tooth loss data used as answered in question #7 and added to the text used the categories (1) No, (2) yes, only one tooth, (3) yes, more than one tooth, (4) yes, all teeth, being used in this way in other studies by Gomes Filho and Verçosa. We insert an information on the studies limitations: “Also, tooth loss was coded according to question of the survey, and it was not possible to use the international classification of functional dentition.” (Lines 323-324).

5. I do not understand why authors opted to maintain ten years (children with mixed dentition) or more in the sample. Joining children, adolescents, adults and older adults in the same analysis. Difficult to interpret

**Response:** We considered important to analyze a wide age range, since we believe that inequality problems affect the population from early stages of life. In this sense, we observed that the associations were consistent and independent of age in the multiple model. We understand the reviewer's concern, however, as we described in the methods, our interviewers were trained and instructed to ask the question about tooth loss excluding teeth extracted for orthodontic reasons, disregarding teeth extracted for orthodontic, wisdom and baby tooth reasons (this information has been added to the text). This can minimize the risk of a classification bias that the age group would have in relation to studies of tooth loss and social conditions. (Lines 96-97).

6. Descriptive analysis need the n and CI 95% (supplemental file 1)
**Response:** Thanks for the suggestion, the data was entered into the table. (Page 17)

7. How was tooth loss collected in the sample? By the number of teeth? Self-report one or more than one? Need more explanation…
**Response:** We added a information in the text: “The outcome variable was self-referred tooth loss, extracted from the question: “Have you ever lost a tooth (upper or lower)? If so, did he lose one, more than one or all of his teeth? (disregard extracted teeth to place braces, wisdom and milk teeth)”, with the options to response: (1) No, (2) yes, only one tooth, (3) yes, more than one tooth, (4) yes, all teeth.” (Lines 90-93)

We also entered the information that the tooth loss was self-reported: “The analysis of the interaction between race and sex in tooth loss was investigated for the first time on a population basis. In this study, information on toothlessness were collected by self-reports…” (Lines 261-262)

8. Need more information on how authors collected data. How many examiners? Calibration? Provide detailed information.
**Response:** We added the following information: “Data were collected from 3021 people aged 10 or over [36], using a pre-coded questionnaire, applied through tablets, by interviewers trained." (Lines 85-87). “When asking about tooth loss, the interviewers informed the respondents to exclude teeth extracted for orthodontic reasons, disregarding teeth extracted for braces, wisdom and baby teeth.” (Lines 96-97)

9. Did the author proceed to sensitivity analysis? "It was verified that the categories of black and brown presented a similar association for tooth loss, in each sex" …and age between browns and blacks? Were they similar or different from Whites?
**Response:** We inform that it was found that the age categories for blacks and browns showed a similar association for tooth loss. This information was inserted in the text: “ It was verified that the categories of black and brown presented a similar association for tooth loss, in each sex and in each age, so these two categories were joined to compose the group of black individuals (Also used by IBGE-Brazilian Institute of Geography and Statistics) [42]. ” (Lines 110-112).

10. Why did the RERI analysis not report the attributable proportion [AP] and synergy index [S]?
**Response:** We appreciate the comment and added the suggested analyzes. In methodology, we included: “As complementary measures to assess the interaction on the additive scale, we calculated the S-index and the attributable proportion (AP). However, because the denominator of the S-index was negative, of those both, we only reported the AP [48], which corresponds to the ratio between the REPI and the prevalence ratio of the category considered as doubly exposed (black women) [48]”. (Lines 188-192). Moreover, in result section, we reported that “According to the AP, 17.17% (95%CI: 2.5% - 31.86%, p=0.02) of the prevalence in black women is due to the interaction”. (Lines 233-234).

11. Tables need to show the n of each category and prevalence and 95% CI, as recommended by Knol & VanderWeele (2012). This will add additional transparency for readers.
**Response:** We appreciate the recommendation. We reviewed the manuscript and verified that the table already followed exactly the orientations of the reference. That is, the number of cases and non-cases in each category, the association measure and its confidence interval. However, we agree that it would be good to report unadjusted prevalence. Therefore, we added the prevalence with its confidence interval in each category. (Page 9)

12. Analyzing absolute prevalence in tooth loss, the percentages (Table 1 ) for white men is 52% ( authors did not put 95% CI), for white women 59% ( why prevalence ratio (PR)under 1?), black man 41% ( lower than white man? ) and black women 53% ( lower than white woman?). ….with slight absolute differences. Problems with the outcome selection as commented in comments #2 and #5 joining children with older adults…

**Response:** Different than unadjusted measures, the estimates of prevalence ratios in this manuscript considered both the sample weights of the complex design of the study and the adjustment for age and IPVS, as informed in the text: “The analyses were conducted using the svy (survey) option of STATA 14.0, which considers the study design weights with complex sampling”. (Lines 177-178). We appreciate the comment, and we agree. So, we added the explanation on the footnote of the table: “PRs are adjusted by IPVS and age, and weighted according to complex sample design”

We reviewed the calculations and confirmed the values. (Page 9).

Reviewer #2:

The topic studied is relevant and well-justified in the Introduction section.
Methods are well-reported and properly designed to answer the research question. A few typos should be revised (e.g., Ln. 92 – the classification “very low” is presented twice).

**Response:** We appreciate the recognition and inform you that the suggestions have been modified in the text. (Line 129).

Data were analyzed adequately, and the results are clearly presented and discussed.
Ln. 178 – Supplementary Figure 1 shows the opposite behavior.

**Response:** In the crude analysis, black men have the lowest tooth loss and among women, black women have less tooth loss compared to white women. However, when we do the adjusted analysis, we find the opposite.

As explained in the text: “In the crude analysis, tooth loss of all teeth was more frequent in women, particularly in white women, and less frequent in black men (Supplementary Figure 1). After adjustments, the prevalence of tooth loss was 19% higher in black women compared to white men (PR: 1.19; 95%CI: 1.05-1.34).” (Lines 217-220)

Reviewer #3:

 I would like to congratulate the authors for their work and willingness to investigate differences between racial minorities and their privileged peers in an outcome that is of great importance in the field of public health. I'm sure the authors will appreciate my comments aimed at providing an outside view on how this manuscript can improve.
**Response:** We appreciate the recognition.

Introduction
1. I would like to ask authors not to use "Blacks". Please use these words only as adjectives, using them as a noun is dehumanizing. Please write "Black individuals", for example.
**Response:** We changed for “black individuals”.

2. In the introduction, the authors present that the main health inequities related to race are: a) due to lesser access to health services; and b) due to socioeconomic issues. However, these are just two of the many factors where race is a cause of health inequities. It is not correct to reduce or omit other factors such as the slavery past of Brazilian society and Latin America. Thus, considering that the authors aim to address the influence of race on tooth loss, it is necessary that the authors deepen the discussion on this topic in the introduction.
**Response:** We appreciate the suggestion and inform you that we have modified the text as suggested. (Lines 25-34).

3. Please provide a very specific reason for what race means and why it is important to study it in the breakdown of oral health outcomes.

**Response:** Thank you. We have included this information in this paragraph of the text: “Racial minorities, in this case black people, may biologically embody the effects of racism [7,12], with everyday (or even less common) discriminatory exposures [12]. Adversities throughout life, such as poverty, psychosocial stresses, stereotypes, and the context of housing, can affect the physical and mental health, altering cardiocirculatory, metabolic and immunological functions [8]. In oral health is possible that inequities are due to poverty [10], levels of education [4] or discrimination in health care [13]. In Brazil, skin color has been studied as proxy of race and this indicator is commonly referred as color/race [14,15,16]. In health, in contrast only to the biologic approach, race may be considered as a concept socially constructed by historic dynamics and power relations [8,12].”

4. I suggest that the authors review the objectives of the study, as in the format it is written, the authors state that there is an association between race and tooth loss. I suggest splitting it into two objectives: a) to investigate a possible association between race and tooth loss and b) assess possible interactions between race and sex in association with tooth loss.
**Response:** We accept the suggestion, and the text was changed lines 64-65.

5.There is also a need for greater attention to standardize the words used throughout the text, such as “race”, “race/skin color”, and “self-reported skin color” to refer to different racial groups in Brazil. In addition to needing to clearly answer, right in the introduction, why to study racial and gender inequities in face of tooth loss, the authors need to review the use of “skin color” throughout the text. If the authors are referring to the Brazilian classification system, it is important to make this clear, and to recognize first that the official wording has been "color/race" since 1991, and probably the same one used in the study. It should be clear that the complexity of the classification is beyond skin color and much more related to how society treats racial minorities, given racism and racial discrimination. I suggest that authors choose only "race" to facilitate understanding by international readers, making this reduction clear from the official classification. Please provide a very specific reason for what race means and why it is important to study it in the breakdown of oral health outcomes.

**Response:** Thank you. We used “race” to define “color/race” and we clarify in the methods section (lines 107-110): “The race variable studied was self-reported. In Brazil this is the official method of racial classification since 1991, based on the individuals’ own perception of the color of their skin. In the present study, race/skin color variable was denominated “race” and categorized as black, brown, and white individuals.”.

In the introduction, we also added an information of use of race as a social construct: “In health, in contrast only to the biologic approach, race may be considered as a concept socially constructed by historic dynamics and power relations [8,12].” (Lines 33-34)

After a paragraph explaining about gender inequalities in tooth loss (lines 59-63)., we include the need and possibility of studying race and gender in intersectional aspects: “The evidence of racial and gender inequalities in tooth loss could bring the subgroup of black women underprivileged in relation to this theme. In this way, it is worth considering the analytical strategy of intersectionality, developed by Crenshaw in 1989 to study the black feminism. Intersectionality, drawing on a set of social issues and perspectives, explain an experience of multiple subordination that cannot be reduced to the simple sum of underprivileged groups [34].”

6. The authors were not able to clearly show in the introduction how the current manuscript has originality compared to other articles already published.
**Response:** We thank you for the note made, to make the originality of the study clear, we added: **“**To our knowledge, there are no studies looking at the relationship between race and sex in relation to tooth loss and this study contributes to filling this gap.” (Lines 66-67).

7. In addition, after reading the introduction, I would like to suggest some references for the authors to read before proceeding with the reformulation of the manuscript:
-rdszz Phelan JC, Link BG. Is Racism a Fundamental Cause of Inequalities in Health? Annu Rev Sociol. 2015;41(1):311-330.
- Bastos JL, Celeste RK, Paradies YC. Racial Inequalities in Oral Health. J Dent Res. 2018:22034518768536.
- Costa F,et al., Racial and regional inequalities of dental pain in adolescents: Brazilian National Survey of School Health (PeNSE), 2009 to 2015. 2021 Cad Saude Publica. 2021 Jun 25;37(6):e00108620.
- Williams DR, Priest N, Anderson NB. Understanding associations among race, socioeconomic status, and health: Patterns and prospects. Health Psychol. 2016;35(4):407-411.
**Response:** We are very grateful for the suggestions and inform you that they were fundamental in making our introduction more grounded.

Methods:
8. In the section of study variables, please, provide all cathegorization of all variables. For example, In which category were yellow and indigenous individuals included?
**Response:** We added an information about yellow and indigenous individuals: “Individuals who declared themselves as yellow, indigenous, or other races were not studied, as they represented less than 2% of the population.” (Lines 113-114).

9. I recommend changing the label from the "black" to "black/brown individuals" since brown individuals were also included in this category.
**Response:** We made the change in the text. (Lines 110-111)

10. Please also present a paragraph supporting the assumptions presented in the DAG.

**Response:** We made the change in the text line 146. “In the DAG, we represented the effects of race and sex on oral health, which can be mediated by income [3,4], education [4] and, more proximally to the outcome, by health behaviors and the dental care [6]. Other variables, such as age, familiarity, patterns of participation in the research, place of residence and IPVS [7], were also considered in the causal diagram (Figure 1). Because it can be a list of unmeasured common causes of both place and race, we follow a convention that visually simplifies the DAG by representing all unmeasured variables with the same causal structure (i.e., the same arrows in and out) as a single node (47). In this case, we included the concept of “familiarity” as that node representing all common determinants of both, place of residence and race. On the other hand, vulnerability (as a context variable measured with the IPVS) in which people are born, would neither determine or be determined by race. But it would be determined by factors such as familiarity and place of residence. The patterns of participation would be a common effect of sex and age, however, even if it was conditioned this would not chance the adjustment set suggested for the DAG. A double-sided broken arrow between sex and race was included to represent the hypothesis of an interaction between race and sex in oral health.” (Lines 149-161)

- how was tooth loss originally collected by DMF-T?

**Response:** We added an information about the original question about tooth loss: “The outcome variable was self-referred tooth loss, extracted from the question: “Have you ever lost a tooth (upper or lower)? If so, did he lose one, more than one or all of his teeth? (disregard extracted teeth to place braces, wisdom and milk teeth)”, with the options to response: (1) No, (2) yes, only one tooth, (3) yes, more than one tooth, (4) yes, all teeth.” (Lines 90-93)

We also entered the information that the tooth loss was self-reported: “The analysis of the interaction between race and sex in tooth loss was investigated for the first time on a population basis. In this study, information on toothlessness were collected by self-reports; ..” (Lines 261-262)

Discussion:
11. Line 265, page 10. “SUS” needs to be written out in full.

**Response:** We appreciate your suggestion and inform you that it has been added to the text line 306.
