# Supplementary material for: Race (black-white) and sex inequalities in tooth loss: A population-based study
Source: PLoS One. 2022 Oct 13;17(10):e0276103. doi: 10.1371/journal.pone.0276103 (PMC9560604; doi:10.1371/journal.pone.0276103)
Supplement: Attachment — Submitted filename: Response to Reviewers.docx [file pone.0276103.s004.docx]

**PONE-D-21-32428R1**

**Race (black-white) and sex inequalities in tooth loss: a population-based study**

**PLOS ONE**

Dear editors

We are submitting the new version of our manuscript. We would like to thank the reviewers for the suggestions, which have certainly enriched our paper. All comments and suggestions were fully taken into account in the preparation of this new version. Our point-by-point responses to the issues raise are listed below:

Review Comments to the Author

1. If the authors have adequately addressed your comments raised in a previous round of review and you feel that this manuscript is now acceptable for publication, you may indicate that here to bypass the “Comments to the Author” section, enter your conflict of interest statement in the “Confidential to Editor” section, and submit your "Accept" recommendation.

Reviewer #2: All comments have been addressed

Reviewer #3: All comments have been addressed

**Response:** We appreciate the comments.

2. Is the manuscript technically sound, and do the data support the conclusions?
The manuscript must describe a technically sound piece of scientific research with data that supports the conclusions. Experiments must have been conducted rigorously, with appropriate controls, replication, and sample sizes. The conclusions must be drawn appropriately based on the data presented.

Reviewer #2: Yes

Reviewer #3: Yes

**Response:** We appreciate the comments.

3. Has the statistical analysis been performed appropriately and rigorously?

Reviewer #2: Yes

Reviewer #3: Yes

**Response:** We appreciate the comments.

4. Have the authors made all data underlying the findings in their manuscript fully available?
The [PLOS Data policy](http://www.plosone.org/static/policies.action#sharing) requires authors to make all data underlying the findings described in their manuscript fully available without restriction, with rare exception (please refer to the Data Availability Statement in the manuscript PDF file). The data should be provided as part of the manuscript or its supporting information, or deposited to a public repository. For example, in addition to summary statistics, the data points behind means, medians and variance measures should be available. If there are restrictions on publicly sharing data e.g. participant privacy or use of data from a third party—those must be specified.

Reviewer #2: No

Reviewer #3: Yes

**Response: The raw data and a codebook were deposited in the public repository as requested.** **Access address:** [**https://doi.org/10.25824/redu/J9YOTW**](https://doi.org/10.25824/redu/J9YOTW) **.**

5. Is the manuscript presented in an intelligible fashion and written in standard English?
PLOS ONE does not copyedit accepted manuscripts, so the language in submitted articles must be clear, correct, and unambiguous. Any typographical or grammatical errors should be corrected at revision, so please note any specific errors here.

Reviewer #2: Yes

Reviewer #3: Yes

**Response:** We appreciate the comments.

6. Review Comments to the Author

Please use the space provided to explain your answers to the questions above. You may also include additional comments for the author, including concerns about dual publication, research ethics, or publication ethics. (Please upload your review as an attachment if it exceeds 20,000 characters)

Reviewer #2: All suggestions done by this reviewer in the first version were adequately addressed. Moreover, accepting the recommendations done by the other reviewer substantially improved the revised version of the manuscript.

**Response:** We appreciate the comments.

Reviewer #3: The present study has improved considerably in this version. I still have a few points to point out.

Skin color self-report should not be a limitation of the study, as this is the best strategy to investigate race in Brazil, since race and skin color can be interpreted as synonyms in the Brazilian context.

**Response:** We agree with the reviewer. We removed the self-declaration race/ skin color from the limitation.

In addition, rethinking from the statements of reviewer #1, I believe that the inclusion of individuals with mixed dentition is really difficult to justify and can imply large biases as well as the way of categorizing tooth loss. The authors mention that they used the question of the study by Gomes Filho and Verçosa, however, they collected the answers differently. This is a critical point of the study.

**Response:** The interviewers did not consider tooth loss those extracted for orthodontic reasons, disregarding teeth extracted for braces, wisdom, and milk (baby) teeth (lines 105-106). Further, regarding the categories analyzed, as described in figure S1, the outcome had an important proportion of people without tooth loss suggesting that a valid and informative approach is to focus on differentiating loss and no loss. Moreover, we also considered the outcome as an ordinal variable resulting in consistent associations (as commented in the discussion, lines 330-336). Therefore, no additional modifications were made regarding this point.

The authos relate “After adjustment for covariates suggested by a directed acyclic graph”; however, is not appropriate describe that DAG “suggest” adjustments. Report only that DAG was used to select covariates.

Response: We appreciate the suggestion has been modified as recommended. Line 18.

7. PLOS authors have the option to publish the peer review history of their article ([what does this mean?](https://journals.plos.org/plosone/s/editorial-and-peer-review-process#loc-peer-review-history)). If published, this will include your full peer review and any attached files.


If you choose “no”, your identity will remain anonymous but your review may still be made public.

**Do you want your identity to be public for this peer review?** For information about this choice, including consent withdrawal, please see our [Privacy Policy](https://www.plos.org/privacy-policy).

Reviewer #2: No

Reviewer #3: **Yes:** Luiz Alexandre Chisini

**Response:** We appreciate the comments.
